# Gene activated adipose tissue fragments as advanced autologous biomaterials for bone regeneration: osteogenic differentiation within the tissue and implications for clinical translation

**DOI:** 10.1038/s41598-018-36283-6

**Published:** 2019-01-18

**Authors:** Bin Ren, Volker M. Betz, Christian Thirion, Michael Salomon, Roland M. Klar, Volkmar Jansson, Peter E. Müller, Oliver B. Betz

**Affiliations:** 1 Department of Orthopedic Surgery, Physical Medicine and Rehabilitation, University Hospital Grosshadern, Ludwig-Maximilians-University Munich, Marchioninistr 15, 81377 Munich, Germany; 2 Sirion Biotech GmbH, Am Klopferspitz 19, 82152 Martinsried, Germany

## Abstract

Cost-effective, expedited approaches for bone regeneration are urgently needed in an ageing population. Bone Morphogenetic Proteins (BMPs) stimulate osteogenesis but their efficacy is impeded by their short half-life. Delivery by genetically modified cells can overcome this problem. However, cell isolation and propagation represent significant obstacles for the translation into the clinic. Instead, complete gene activated fragments of adipose tissue hold great potential for bone repair. Here, using an *in-vitro* culture system, we investigated whether adenoviral transduction with human BMP-2 can promote osteogenic differentiation within adipose tissue fragments. Osteoinduction in adipose tissue fragments was evaluated by quantitative reverse transcriptase polymerase chain reaction, immunohistology and histomorphometry. BMP-2 transduced adipose tissue synthesized BMP-2 protein over 30 days peaking by day six, which significantly promoted osteogenic differentiation as indicated by increased calcium depositions, up-regulation of bone marker genes, and bone-related protein expression. Our results demonstrate that cells within adipose tissue fragments can differentiate osteogenically after BMP-2 transduction of cells on the surface of the adipose tissue. BMP-2 gene activated adipose tissue represents an advanced osteo-regenerative biomaterial that can actively contribute to osteogenesis and potentially enable the development of a novel, cost-effective, one-step surgical approach to bone repair without the need for cell isolation.

## Introduction

Orthopedic, plastic and maxillofacial surgeons experience many clinical conditions requiring the stimulation of bone growth. It is expected that the demand for bone engineering technologies will increase as the longevity of life leads to an ever growing aging population suffering from osteoporosis and diabetes^1,2^.

Adipose tissue is an abundant source of adult mesenchymal stem cells with emerging promise in the field of tissue engineering and regenerative research. The stromal vascular fraction (SVF), in particular, is a heterogeneous collection with progenitor activity that includes mesenchymal stem cells, preadipocytes, pericytes, endothelial cells, immune cells and other stromal components^3^. A putative stem cell population within this SVF was first identified by Zuk *et al*. and named “processed lipoaspirate (PLA)”cells^4,5^. During the past decade, there has been much discussion regarding adipose-derived stem cells (ADSCs) as a form of cell-based therapy in regenerative medicine. However, the nature of a tissue-based response of the SVF or other fractions of adipose tissue under a specific stimulus is still unknown.

The formation of new bone within large critical sized defects still remains a challenge with little progress having been made to develop new and alternative treatment systems that can replace the still golden standard used clinically, the autogenous bone graft^6–8^. Ever since the discovery of bone morphogenetic proteins (BMPs) and their description at initiating bone formation, few clinically relevant applications have been developed especially when regenerating large defects in humans^9,10^. The application of recombinant osteogenic growth factors in the induction of new bone formation at the site of large bone defects continues to be problematic^11^. The short half-life of recombinant BMP has been suggested as the main factor for its low efficacy by preventing continued activation of the osteogenic transduction pathways critical for the modulation of new bone formation^12^. Massive amounts of exogenous proteins are then generally applied to compensate for the half-life effect of the BMPs, which may improve the bone formation quantity but frequently leads to unwanted and dangerous side effects such as tissue edema, seroma, dysphagia, and ectopic bone formation^13,14^.

Gene transfer techniques have emerged as a viable alternative to excessive BMP dosages as they can greatly reduce the amounts of growth factor needed and thereby minimize the number of adverse events^15^. Gene transfer technologies allow sustained delivery of small, more physiological amounts of BMPs providing a long-lasting bone regeneration stimulus. Whilst various gene therapy techniques have been developed ever since its discovery in 1972^16^, the most promising approach for tissue repair so far is the *ex vivo* method^17–19^. This procedure involves the transfer of exogenous cDNA to prior isolated cells *in vitro* and the subsequent implantation of the genetically modified cells back into a tissue lesion. However, because this mode of gene therapeutics is very complex to perform and highly cost ineffective^20^, novel expedited *ex vivo* gene therapy strategies need to be investigated.

One approach that has been shown to be of benefit, as pioneered by Betz *et al*.^20,21^, is the use of gene activated muscle tissue grafts for *in vivo* experiments. In a series of systematic studies, it has been shown that adenoviral gene activated muscle tissue grafts directly result in biological compatible bone defect regenerative implants, mimicking the bone induction principle, that induce substantial new bone formation within osseous lesion sites^21–24^. Subsequently, adipose tissue grafts have also been shown, when transduced with human *BMP-2* (h*BMP-2*), to cause rapid structural and functional healing of large segmental bone defects in rats^25^. The advantages of using gene activated adipose tissue grafts instead of isolated cells are that time-consuming and costly cell culture and expansion can be avoided. Whilst of great interest for the bone regeneration field, the exact mechanism of bone formation by adipose tissue grafts with *hBMP-2* gene activated products remains unclear. Can an osteogenic environment stimulate an osteogenic response within adipose tissue fragments and can the transduction with the *hBMP-2* gene enhance the osteogenic response within the adipose tissue or does the transduced tissue merely serve as a morphogen reservoir generating osteogenic growth factors, which then stimulate the migration and osteogenic differentiation of cells from the surrounding tissue of a bone defect? If the role of the adipose tissue in bone repair was limited to growth factor production and stem cell recruitment from the environment of the lesion, it would make sense to focus on the development of improved drug delivery systems rather than cellular implants and therapeutics.

Hence, the goal of this study was to explore the effect of *hBMP-2* transduction on fragments of subcutaneous adipose tissue from rats *in vitro*, characterize the *hBMP-2* protein expression profile after the application of the adenoviral vector to tissue fragments and evaluate whether bone induction within the transduced adipose tissue can be elicited. By better defining this process, results from this study could provide deeper insight into the repair mechanisms using gene-activated tissue grafts and contribute to the development of new expedited gene therapy approaches facilitating endogenous bone repair.

## Results

### Efficiency of adenoviral particle transduction of adipose tissue

**Figure 1:**
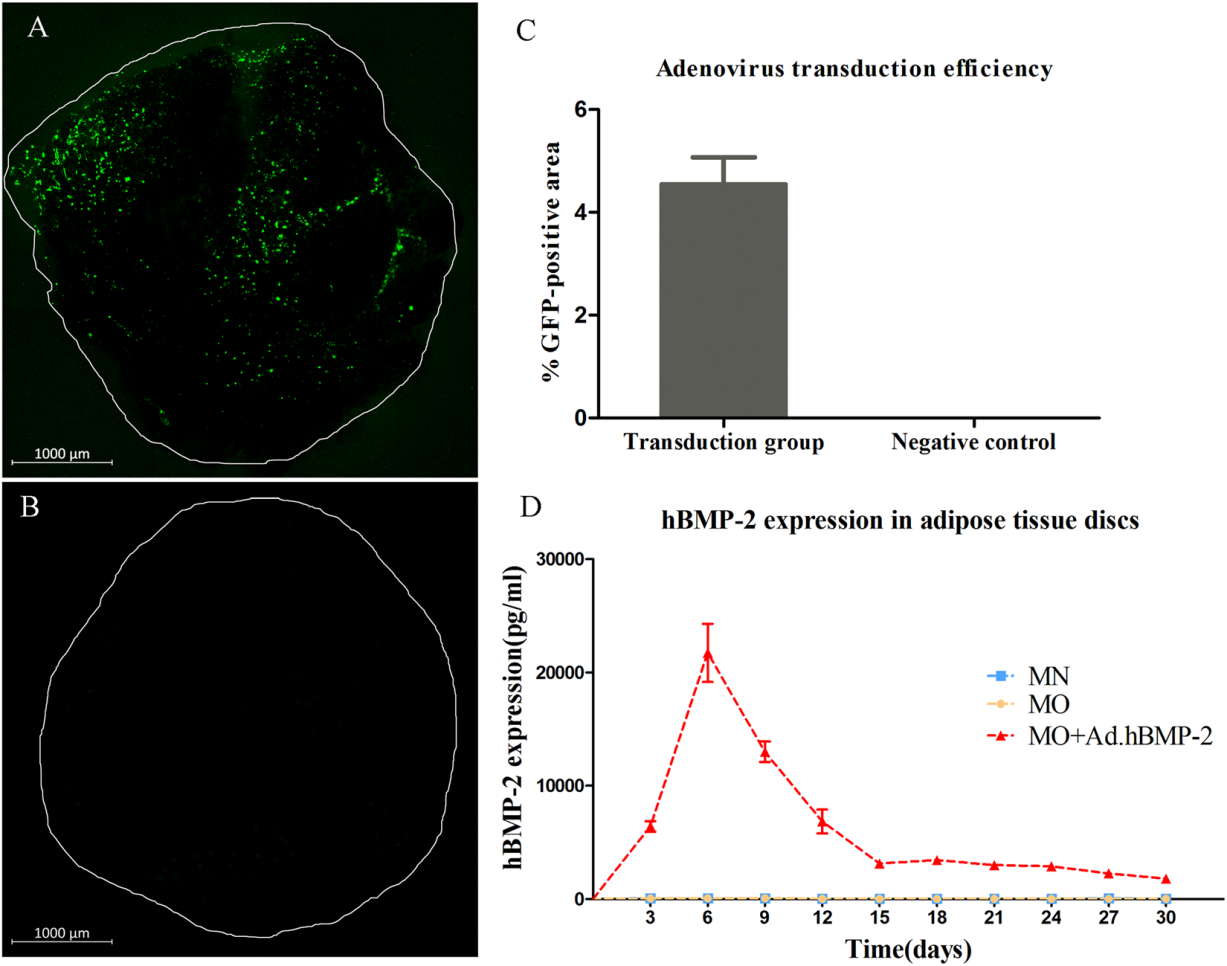
Adenoviral gene transduction efficiency and hBMP-2 protein expression analysis. (**A**) Confocal fluorescent microscopy showing the transduction efficiency of Ad.GFP in 4 mm diameter adipose tissue discs. Cells residing on the surface of the adipose tissue fragments were transduced by Ad.GFP and showed detectable positive green signals. (**B**) Green fluorescent signals were undetectable in non-transduced tissue discs (negative control). (**C**) Quantitative analysis of transduction efficiency indicated that 4.56 ± 0.51% area of transduced adipose tissue discs was transduced as GFP-positive, while 0% area of negative control tissue discs. Nine samples per group were used for the analysis. (**D**) hBMP-2 protein expression from media harvests every 3 days intervals until day 30. The protein synthesis level in Ad.hBMP-2 transduced tissue discs cultured in osteogenic medium (MO + Ad.hBMP-2) showed a significant increase from day 3 to day 15, and then decreased gradually until settling at a constant synthesis level from day 15 to day 30. No detectable hBMP-2 was found in non-transduced tissue discs cultured in normal medium (MN) and in osteogenic medium (MO). Nine samples per group were used for ELISA and measurements were performed in triplicate.

Transduction of each adipose tissue disc with 1 × 10^8^ IU Ad.GFP revealed extensive infection 24 hours after infection (Fig. 1A). Cells residing on the surface of the adipose tissue fragments were transduced by Ad.GFP and showed detectable positive green signals. Comparatively, green fluorescent signal was not detected in the entire fragment of non-transduced adipose tissue (Fig. 1B). The quantitative analysis calculated from nine specimens shows that 4.56% ± 0.51% area of tissue disc surface was GFP-positive (Fig. 1C). Little to no signals could be detected within deeper areas of tissue, since the transduction requires a direct attachment between virus particles and cells.

### hBMP-2 synthesis after Ad.hBMP-2 transduction

The supernatants of culture media were collected every third day after initial transduction with 1 × 10^8^ IU of Ad.hBMP-2 to monitor hBMP-2 synthesis over the 30 day culture period. hBMP-2 protein synthesis after transduction in adipose tissue discs significantly increased after day 0, with protein translation of hBMP-2 being highly expressed at day 6 (21736 pg/ml ± 2572 pg/ml) of tissue incubation. The concentration of hBMP-2 was then observed to significantly and seemingly gradually decrease, following tissue culturing after day 6, with protein expression levels settling at a relatively low but constant expression level (2500–3000 pg/ml) from day 15 until day 30. In contrast, non-transduced adipose discs (MN = normal medium and MO = osteogenic medium), as expected, did not show any detectable hBMP-2 secretion. (Fig. 1D)

### Gene expression patterns

**Figure 2:**
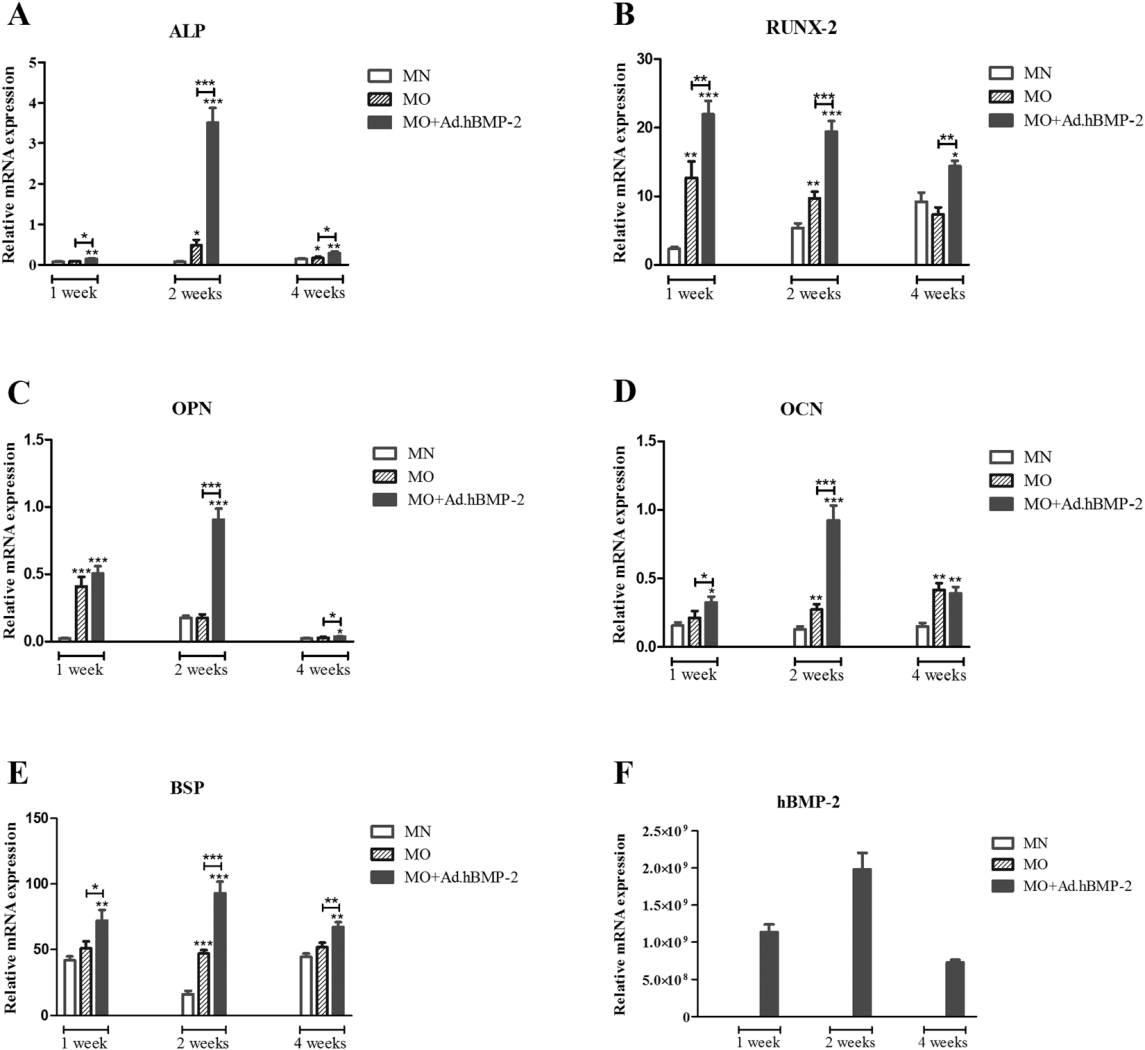
Quantitative comparisons of bone marker gene expressions between MN (normal medium), MO (osteogenic medium) and MO + Ad.hBMP-2 groups. *ALP* (**A**), *RUNX-2* (**B**), *OPN* (**C**), *OCN* (**D**) and *BSP* (**E**) gene expressions were significantly increased in MO + Ad.hBMP-2 group when compared to MO and MN, whilst significant differences were also found between MO and MN at most time points. h*BMP-2* (**F**) was only expressed in Ad.hBMP-2 transduced group during the culture period, of which the highest gene expression was at 2 weeks. Four tissue discs cultured in each well were harvested as one sample for RNA isolation, and 9 samples per group were used for qRT-PCR. The significance level of MO + Ad.hBMP-2 *vs*. MN as well as MO *vs*. MN, was marked on the top of column without capped line (*for p < 0.05, **for p < 0.01 and ***for p < 0.001).

After 1 week or 2 weeks of culture, osteogenic gene markers *ALP* (Fig. 2A) and *RUNX-2* (Fig. 2B) in the MO + Ad.hBMP-2 group were quickly promoted, peaking at 2 weeks and 1 week correspondingly, significantly higher than in the other two groups. Although expression values declined after the second week, they were still significantly higher than in the MO and MN groups. The highest *OPN* and *OCN* gene expressions were detected in the MO + Ad.hBMP-2 group at 2 weeks (Fig. 2C,D), while *BSP* was continuously expressed at a high and relatively stable pattern throughout the culture period (Fig. 2E). These markers were all significantly upregulated as compared to MN and MO groups. Significant differences between the MO group and the MN group were also detected at most time points.

hBMP-2 protein expression pattern, as quantified by ELISA, was confirmed by qRT-PCR. h*BMP-2* gene expression was highly promoted after transduction during the whole culture period, peaking around the second week. There was almost no detectable h*BMP-2* gene expression in non-transduced rat adipose discs **(**Fig. 2F**)**.

### Calcium deposition within adipose tissue discs and quantitative analysis

**Figure 3:**
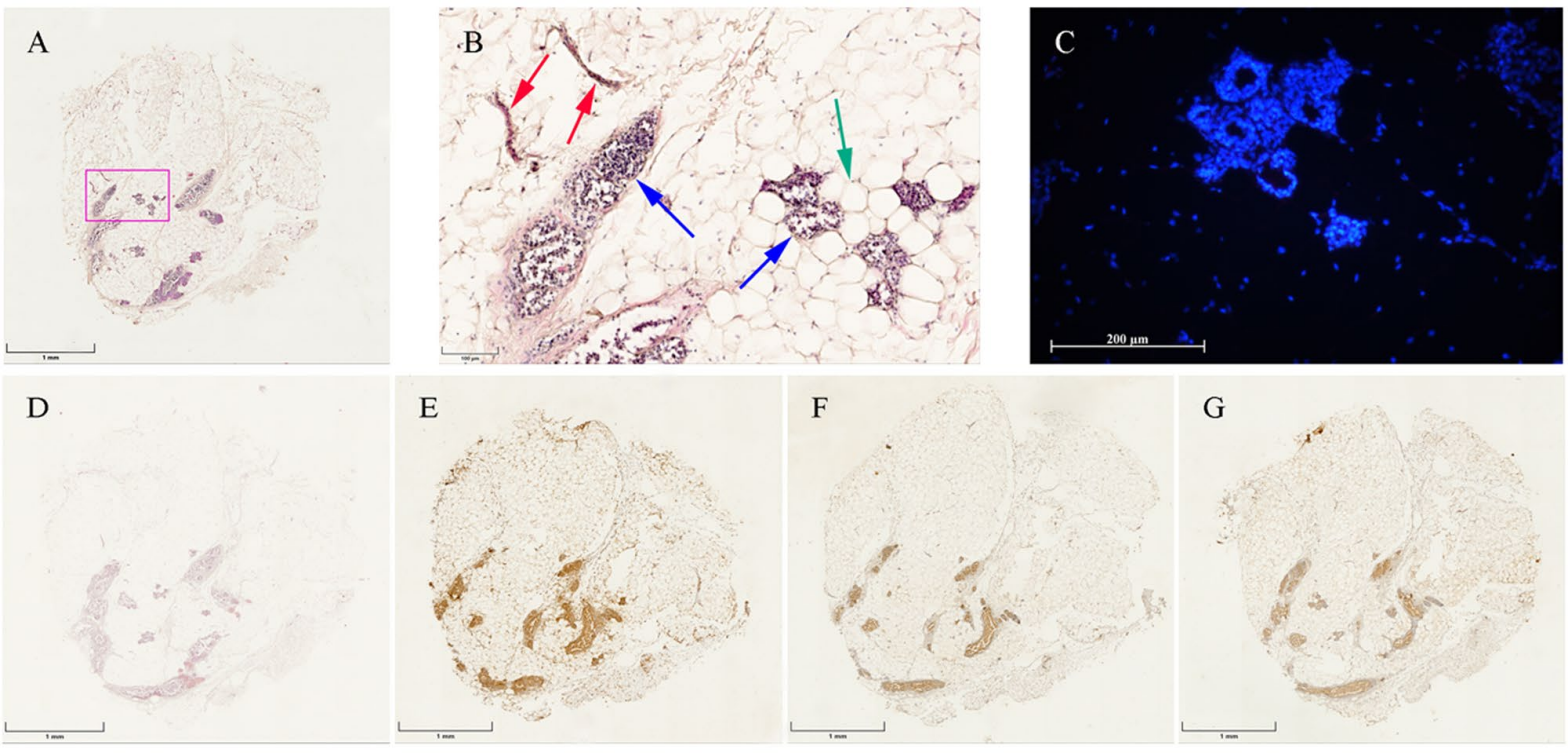
Histological staining of untreated fresh adipose tissue discs. (**A**) (×2.5); (**B**) (×15): HE staining showing the structure of adipose tissue consisting of adipocytes (green arrow) and stromal vascular fraction (SVF) (red and blue arrows), which contains vascular tubes (red arrows) and a cellular complex including pre-adipocytes, mesenchymal stem cells, endothelial progenitor cells etc. (blue arrows). (**C**) Immunofluorescence of hBMP-2 revealing hBMP-2 was undetectable in untreated rat adipose tissue. (**D**) Alizarin red S staining showing calcium deposition was absent in untreated fresh adipose tissue. Immunohistochemistry of OCN (**E**), OPN (**F**) and Scl (**G**) indicating the co-localization of minimally endogenously expressed proteins of OCN, OPN and Scl in fresh adipose tissue, respectively.

Alizarin red S staining was used to identify calcium deposits containing osteocytes, where calcium deposits are stained bright red. Of note, for the untreated fresh adipose tissue, alizarin red S staining was negative (Fig. 3D), indicating that calcium does not exist in untreated adipose tissue.

**Figure 4:**
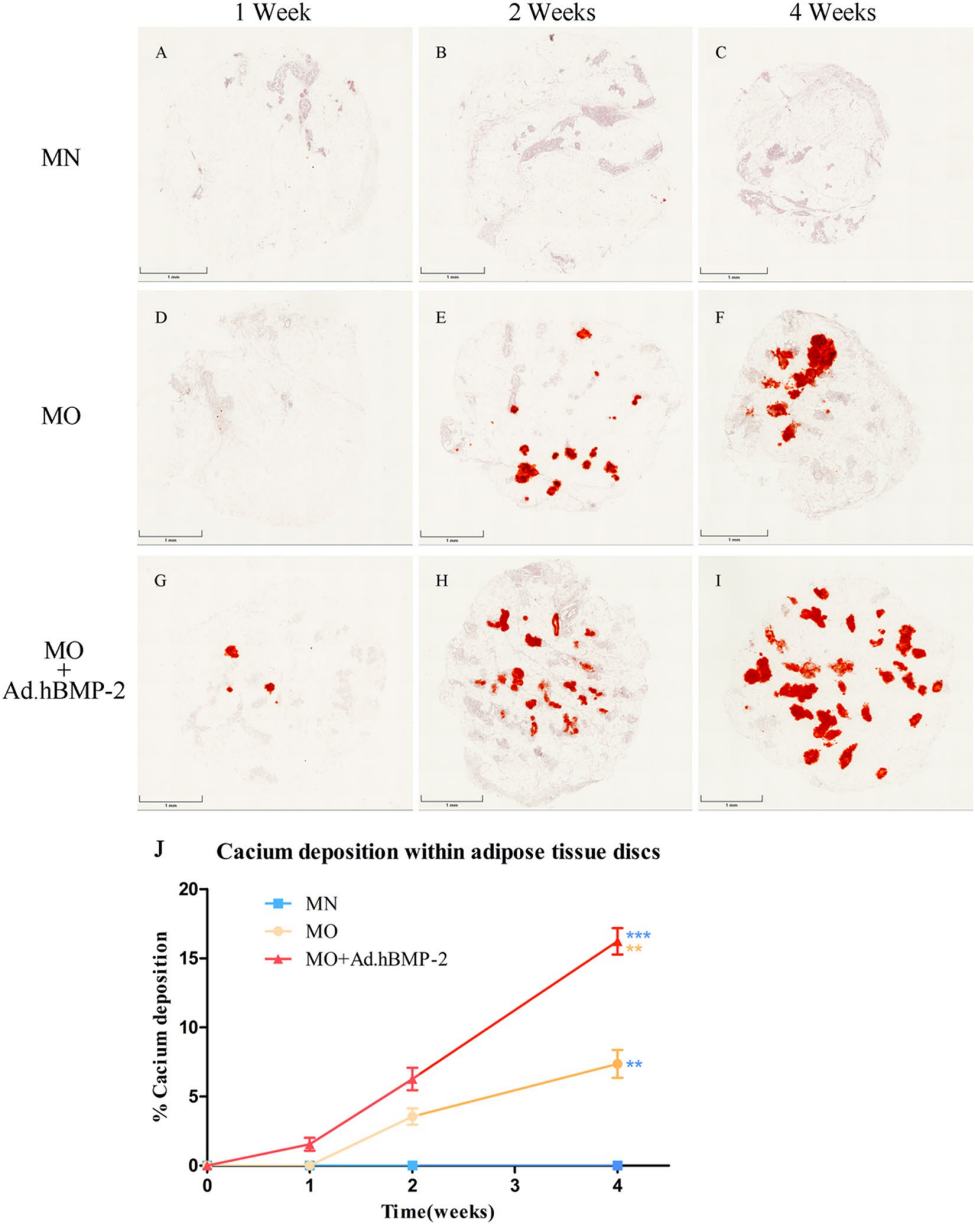
Alizarin red S staining of adipose tissue discs cultured in MN, MO and MO + Ad.hBMP-2 groups after 1, 2 or 4 weeks of culturing. Bright red stained calcium could be clearly seen in MO + Ad.hBMP-2 and MO groups. Sections were presented as: MN: (**A**) 1 week, (**B**) 2 weeks, (**C**) 4 weeks; MO: (**D**) 1 week, (**E**) 2 weeks, (**F**) 4 weeks; and MO + Ad.hBMP-2: (**G**) 1 week, (**H**) 2 weeks, (**I**) 4 weeks. Scale bar = 1 mm. (**J**) Histomorphometric analysis of calcium depositions within the adipose tissue discs from MO + Ad.hBMP-2, MO and MN groups. Significantly higher calcium depositions were observed in MO + Ad.hBMP-2 compared to MO (**) or MN (***); calcium content of MO was significantly higher than that of MN (**); MN showed little to no calcium deposits in the tissue discs. One representative section per tissue sample was analyzed. Nine tissue samples per group/condition were used for histomorphometry.

Throughout the 4 weeks culture period, MN group did not display any calcium depositions (Fig. 4A–C). After only 1 week of culturing, the MO + Ad.hBMP-2 group had started to show some tiny areas with positive staining (Fig. 4G). After 2 and 4 weeks of culturing, samples in the MO (Fig. 4E,F) and MO + Ad.hBMP-2 groups (Fig. 4H,I) displayed gradually increased calcium deposits, which were primarily restricted to the stromal-vascular fractions of the adipose tissue disc. Comparatively, the adipose tissue discs treated with MO + Ad.hBMP-2 possessed significantly larger areas of calcium deposition. The quantitative analysis showed that after 4 weeks of culture in MO + Ad.hBMP-2, calcium deposition had expanded across 15.21% ± 1.12% area of the adipose tissue disc, which was a significant increase when compared to MO (7.36% ± 1.01%) and MN (0%) groups (Fig. 4J).

### Immunohistochemistry and quantitative analysis

Immunohistochemical staining of OCN, OPN and Scl were detected in the histological sections of fresh untreated adipose tissue discs and all treated groups cultured for 1, 2 and 4 weeks. In the untreated specimens, OCN, OPN and Scl (Fig. 3E–G) were minimally endogenously expressed and were primarily found in the stromal vascular fractions of the adipose tissue, but absent in the adipocytes.

**Figure 5:**
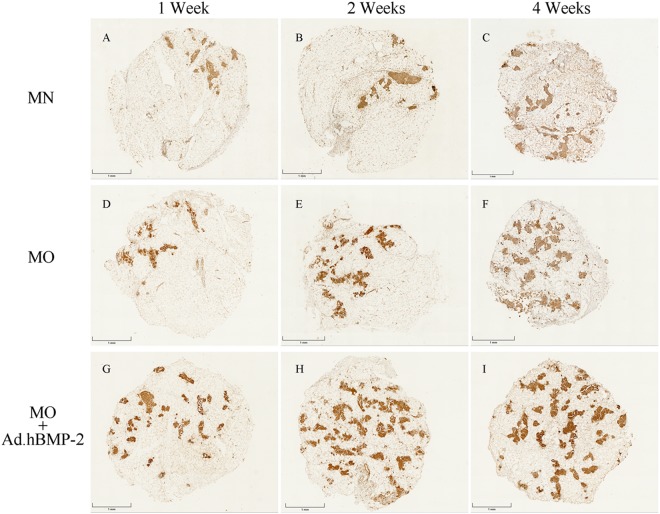
Immunohistochemical staining of osteocalcin (OCN) for adipose tissue discs cultured in MN, MO and MO + Ad.hBMP-2 groups after 4 weeks of culturing. MN: (**A**) 1 week, (**B**) 2 weeks, (**C**) 4 weeks; MO: (**D**) 1 week, (**E**) 2 weeks, (**F**) 4 weeks; and MO + Ad.hBMP-2: (**G**) 1 week, (**H**) 2 weeks, (**I**) 4 weeks. MO + Ad.hBMP-2 group showed the most intensive staining at all the time points. The sections showing staining for osteocalcin, osteopontin (Fig. 6), and sclerostin (Fig. 7) were retrieved from the same representative specimens and the same time points from in adjacent positions in order to detect their co-expression. Scale bar = 1 mm.

**Figure 6:**
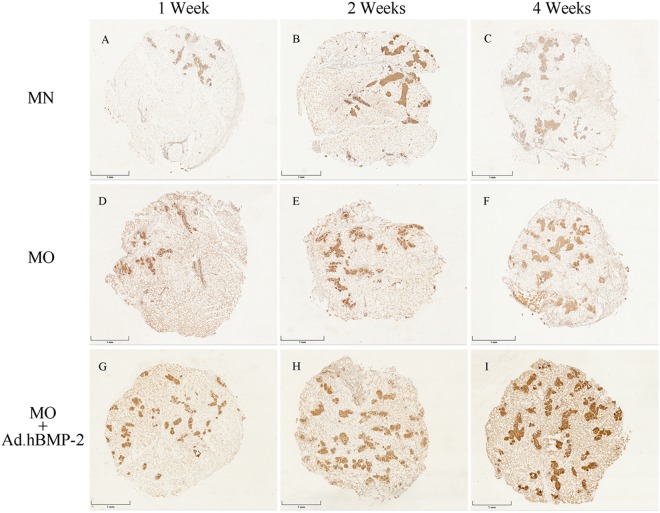
Immunohistochemical staining of osteopontin (OPN). MN: (**A**) 1 week, (**B**) 2 weeks, (**C**) 4 weeks; MO: (**D**) 1 week, (**E**) 2 weeks, (**F**) 4 weeks; and MO + Ad.hBMP-2: (**G**) 1 week, (**H**) 2 weeks, (**I**) 4 weeks. The most intensive staining was presented in MO + Ad.hBMP-2 group at each time point.

**Figure 7:**
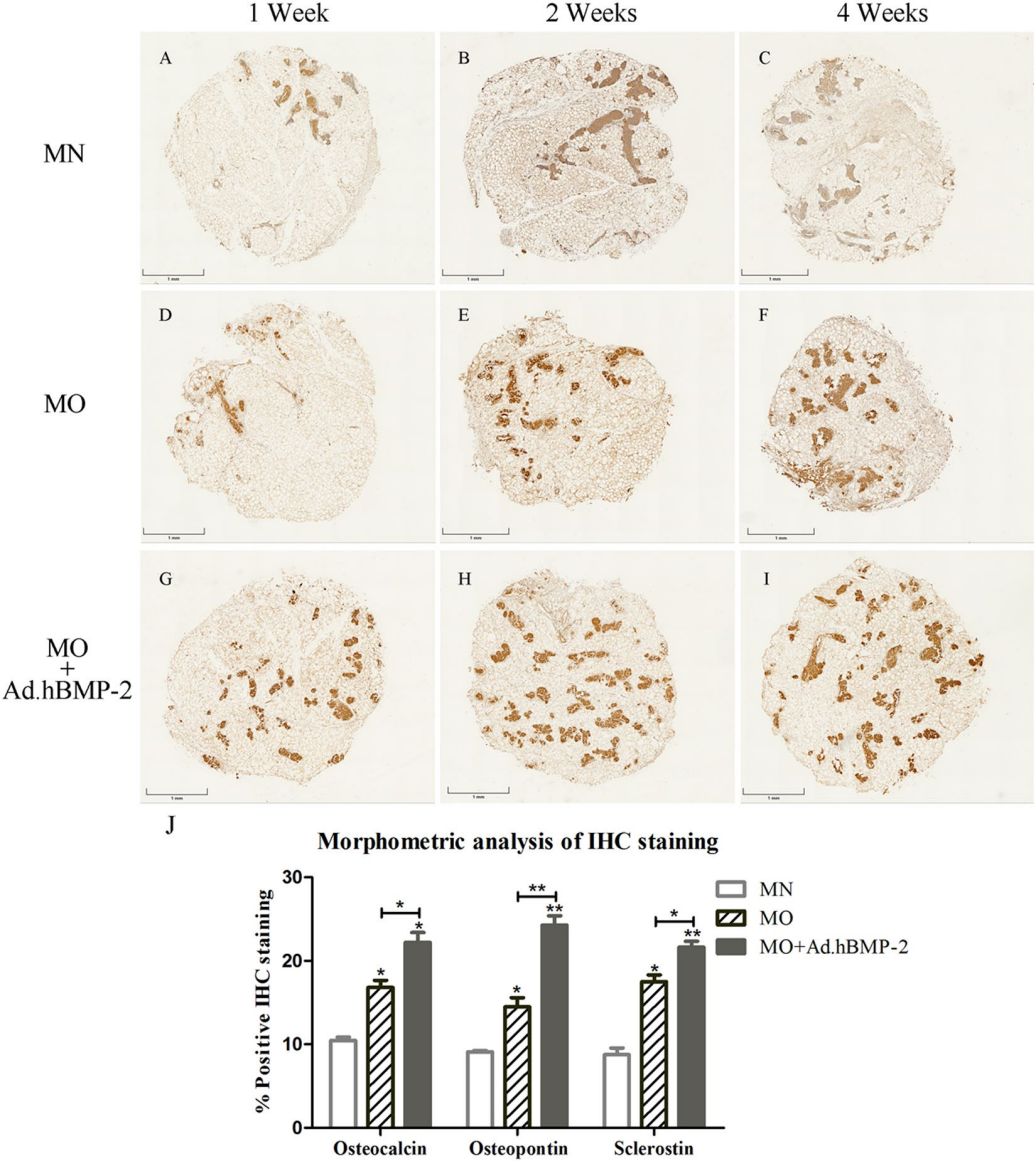
Immunohistochemical staining of sclerostin (Scl) and morphometric analysis of the IHC staining products: OCN, OPN and Scl. MN: (**A**) 1 week, (**B**) 2 weeks, (**C**) 4 weeks; MO: (**D**) 1 week, (**E**) 2 weeks, (**F**) 4 weeks; and MO + Ad.hBMP-2: (**G**) 1 week, (**H**) 2 weeks, (**I**) 4 weeks. The most intensive staining was presented in MO + Ad.hBMP-2 group at each time point. (**J**) Morphometric analysis of the IHC staining products: OCN, OPN and Scl at four weeks. The largest staining areas of OCN, OPN and Scl were observed in the sections from MO + Ad.hBMP-2 group, followed by MO, whilst MN the smallest. One representative section per tissue sample was analyzed. Nine tissue samples per group/condition were used for morphometric analysis. The significance level of MO + Ad.hBMP-2 *vs*. MN as well as MO *vs*. MN, was marked on the top of column without capped line (*for p < 0.05, **for p < 0.01 and ***for p < 0.001).

In the experimental groups, OCN (Fig. 5A–I), OPN (Fig. 6A–I) and Scl (Fig. 7A–I) were detected expressed at different intensities. The staining in MO and MO + Ad.hBMP-2 groups were continuously increasing during the 4 weeks culture period, especially in the MO + Ad.hBMP-2 group. In contrast, the changes in the MN group were not as obvious as in other two groups.

The proportion of positively stained IHC product in the whole tissue disc was subsequently quantified by morphometric analysis (Fig. 7J). The MO + Ad.hBMP-2 group showed a significantly more intensive staining in comparison with MN or MO group after 4 weeks of incubation. Sections in the MO group showed significantly stronger staining signals for OCN, OPN and Scl as compared to MN group. Considering that sections of the same time point for the three immunohistochemical antigens were from the same specimens, OCN, OPN and Scl were proved to be co-expressed in the same area.

### Immunofluorescence detection of BMP-2 expression

**Figure 8:**
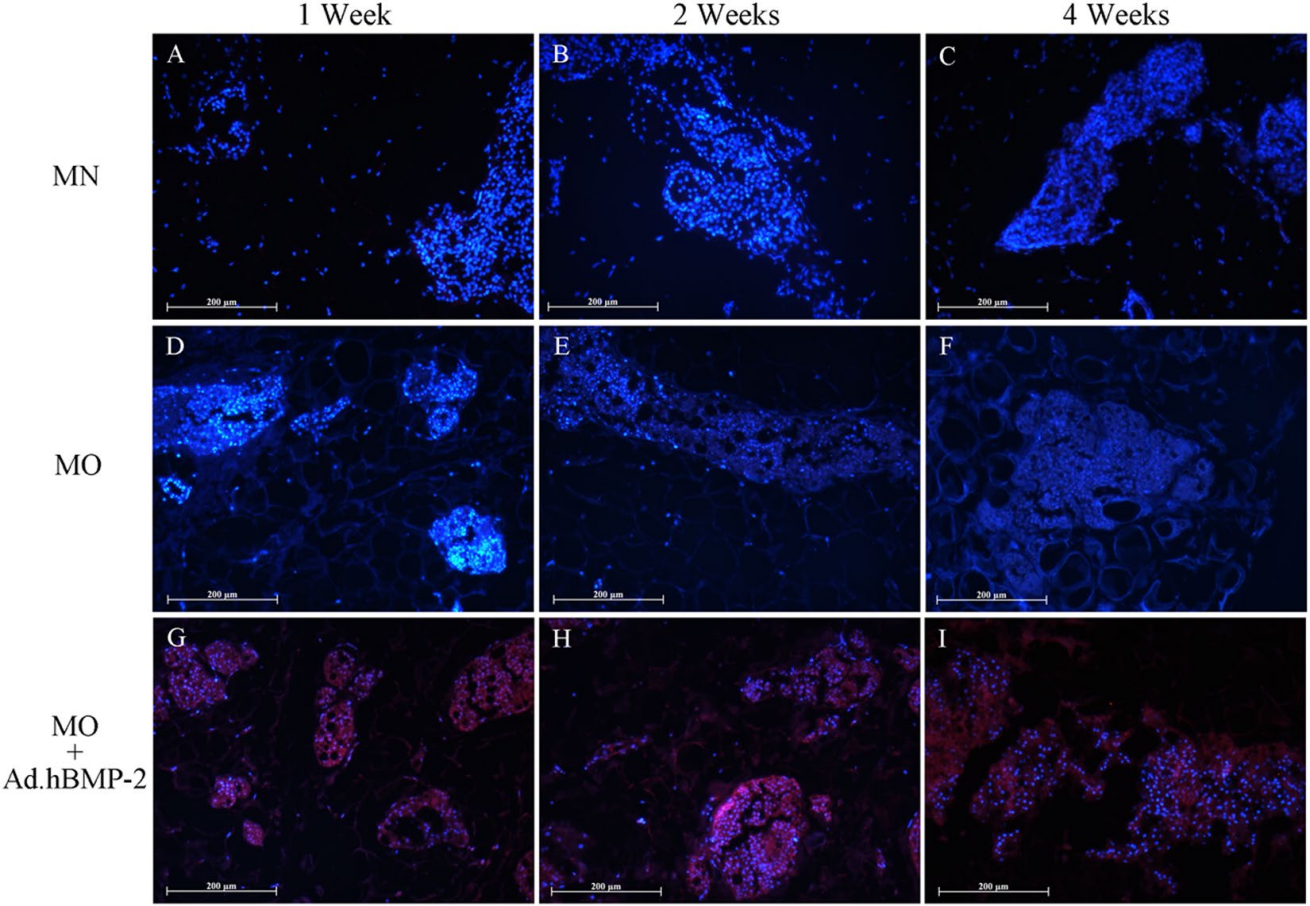
Immunofluorescent staining of hBMP-2. MN: (**A**) 1 week, (**B**) 2 weeks, (**C**) 4 weeks; MO: (**D**) 1 week, (**E**) 2 weeks, (**F**) 4 weeks; and MO + Ad.hBMP-2: (**G**) 1 week, (**H**) 2 weeks, (**I**) 4 weeks. Immunofluorescent staining products of hBMP-2 were shown in red, and nuclei of cells were shown in blue. MO + Ad.hBMP-2 group showed strong red fluorescent for hBMP-2 signals induced by Ad.hBMP-2 transduction, whilst little to no red signals were detected in non-transduced MN and MO groups. Scale bar = 200 μm.

The hBMP-2 was found to be absent in freshly harvested untreated adipose tissue samples (Fig. 3C) as well as in the 4 weeks cultured non-transduced tissue from MN (Fig. 8A–C) and MO groups (Fig. 8D–F). In contrast, immunofluorescent signals could be detected over the whole culture period in the MO + Ad.hBMP-2 group (Fig. 8G–I). The most intensive signals were observed at 1 and 2 weeks, after which there was a loss in signal by the 4th week. These results were consistent with those from hBMP-2 protein and gene expression patterns from ELISA and qRT-PCR respectively.

## Discussion

A significant amount of research has been conducted in the field of bone regeneration. However, the majority of those approaches will not translate into viable clinical applications. Therefore, alternative approaches need to be developed. Gene therapy has emerged as a powerful treatment modality for the regeneration of bone. Instead of utilizing biomaterials combined with morphogens that have so far fallen short of achieving the necessary bone formation clinically^26–28^, genetically altered tissues or stem cells might have the potential to transform directly into the designated tissue types.

Previous studies have indicated that, for example, muscle tissue can be harvested, genetically modified through gene transduction and subsequently heal bone defects within a rat model^21,22^. The present results indicate that much like muscle tissue, adipose tissue can also be sufficiently transduced and express a designated protein product. This was confirmed by immunofluorescent staining of hBMP-2 protein. The qRT-PCR data revealed that mRNA expression patterns of the bone markers *ALP*, *RUNX-2*, *OPN*, *OCN* and *BSP* increased under osteogenic culture conditions. Moreover, the stimulation by the expressed h*BMP-2* led to an earlier and stronger osteogenic response than osteogenic medium alone as indicated by the immunohistochemical staining for OCN, OPN and Scl.

Although the transduction efficiency was relatively low, the amount of transfected cells was adequate to upregulate the hBMP-2 expression to a level that allowed significant osteogenic differentiation within adipose tissue. However, improved BMP-2 delivery systems still need to be explored to further increase osteoinduction and enhance the ability of adipose tissue grafts to regenerate bone defects. One potential way of improving the implant is to transduce a larger number of cells, for example cells in the deeper areas of the adipose tissue fragment. However, if viral vectors are injected into the deeper areas of the tissue pieces, it will not be possible to wash off the vector particles after transduction and prior to implantation. Instead, free vector particles would be introduced into the body and the approach wouldn´t be a true *ex-vivo* gene therapy method with its advantages in terms of safety. Hence, to optimize the approach it might make more sense to focus on advanced vector technologies to improve transduction of cells on the surface of the tissue fragment.

Adipose tissue represents a complex tissue in terms of its cellular composition, as it includes mature adipocytes and other various cell types comprising the stromal-vascular fraction (SVF)^28^. The SVF consists of a heterogeneous mesenchymal population of cells that includes plenty of stem and progenitor cells, and also various differentiated cell types like endothelial cells, erythrocytes, fibroblasts, lymphocytes, monocyte/macrophages and pericytes^29^. Interestingly, our study revealed that calcium seems to be primarily produced within the SVF, showing that the progenitor cells responsible for osteogenic differentiation cells are located within the SVF. However, calcium accumulation was absent in the SVF when the non-transduced adipose tissue was cultured in normal, non-osteogenic growth medium. Our previous study revealed that calcium deposition was also undetectable in hBMP-2 gene activated adipose tissue cultured in normal growth medium, although similar amounts of hBMP-2 protein were produced as compared to the present study^30^. These data confirmed that the progenitor cells within the SVF require the presence of osteogenic medium for osteoinduction in order to initiate and modulate the differentiation process^31^. It has also been shown, that adipose tissue derived stromal vascular fraction can undergo osteogenic differentiation *in vivo* when these progenitor cells were isolated^32^. Our results confirm this observation and we were able to additionally show that osteogenic differentiation of the stromal vascular fractions can be achieved without prior isolation from the adipose tissue. In previous *in vivo* studies, we demonstrated that adipose tissue fragments transduced with BMP-2 in the same way as the adipose fragments observed in the present study were able to heal large bone defects in rat femora^25^. Therefore, we speculate that the osteogenic response of the stromal vascular fractions within adipose tissue fragments can be sufficient to contribute to osseous repair *in vivo* when implanted within a bone defect.

Facilitated endogenous repair based on the implantation of complete soft tissue grafts is an evolving technology showing success in a number of preclinical studies^21–25^. These experiments demonstrated that biological, osteo-regenerative grafts can be generated through genetic modification of cells in these tissue fragments. Besides adipose tissue, muscle tissue has been proven to be an effective implant for bone regeneration^21,23^. In the case of muscle tissue, it is not surprising that osteoinduction can occur within the tissue as this has been demonstrated by the genetic disease *fibrodysplasia ossificans progressiva*^33,34^. Additionally, from patients receiving hip prostheses or individuals suffering from severe soft tissue injuries it is known that bone can form ectopically in muscle tissue^35,36^.

In contrast to muscle tissue, adipose tissue has not been such an obvious candidate tissue for use as an osteo-regenerative implant. Only isolated adipose derived stem cells have received widespread attention with respect to bone regeneration^37–39^. It has been demonstrated that the implantation of adipose derived stem cells, engineered to produce osteogenic molecules, can lead to the healing of large bone defects in various animal models^12,40,41^. Although such *ex vivo* gene therapy approaches involving adipose tissue derived cells have been pre-clinically applied, their implementation within a clinical setting remains a challenge because of high costs and the complex nature of the procedures. The need for two separate surgical procedures and long-term cultivation under good manufacturing practice (GMP) conditions prevents a widespread clinical application of such cell-based therapies^20^. Moreover, holding cells in culture over several days or weeks can increase the risk of contamination and unwanted changes of cell characteristics^42,43^. For these reasons, so called “same day” *ex vivo* gene therapy approaches are currently being investigated, where cells were isolated and genetically modified on the same day of surgery before being implanted within a femoral bone defect to contribute to tissue regeneration^44^.

Alternatively, gene activated adipose tissue fragments could be more beneficial when it comes to healing bone lesion during a single surgical procedure than isolated cells with the “same day” approach, as cellular separations are considered “more than minimally manipulated”^45^. In general, subcutaneous adipose tissue is considered to be an excellent cell source for tissue engineering purposes since it is easy to harvest in large quantities and associated with low donor site morbidity^46,47^. Plastic and reconstructive surgeons have been using adipose tissue for many years as dermal fillers and for rejuvenation and augmentation procedures^48–50^. For bone engineering, the combination of BMPs as osteoinductive molecules and adipose tissue fragments as stem cell containing, biological scaffolds, could be a powerful new source by which bone formation can be accomplished. This is clearly reiterated in the present study results, where the transduction of adipose fragments leads to production of hBMP-2, providing a long-lasting, effective stimulus for osteoinduction within the tissue. Nevertheless, certain questions still need further elucidation by future studies, such as further identification of cells within adipose tissue involved in the osteogenic differentiation and whether the SVF also possesses the potential to regenerate other tissues similar to isolated ADSCs.

In conclusion, we have demonstrated that hBMP-2 transduced adipose tissue fragments can directly transform into tissue with bone-like characteristics within the stromal, multipotent and hematopoietic cell populations of the SVF without prior cell isolation. Our results suggest, that hBMP-2 gene transduced fat tissue particles have the potential to become a clinically applicable treatment option, due to its simplicity and safety features, and could therefore meet the increasing demand for the regeneration of bone tissue in patients.

## Materials and Methods

### Study design

The overall objective of the study was to develop an expedited, cost-effective bone repair method based on the use of adipose tissue fragments instead of commonly used isolated ADSCs. We explored the ability of non-isolated cells within subcutaneous adipose tissue from syngeneic Fischer rats to differentiate osteogenically. The transduction efficiency was confirmed under laser-scanning confocal microscopy and quantified by ELISA, and osteogenic differentiation was evaluated by qRT-PCR, histology, and immunohistochemistry. ELISA and histological analysis were performed in a blinded fashion, whenever feasible.

This study was approved by the responsible committees of the Ludwig-Maximilians-University Munich and the state of Bavaria, Germany. All experiments were performed in accordance with relevant guidelines and regulations.

### Tissue harvest

Adipose tissue was harvested from 6 donor Fischer rats (F-344/DuCrl). Only subcutaneous fat from the inguinal depot was collected to maintain the homogeneity of the tissue source. The harvested tissue was placed in a petri dish with DMEM/Ham’s F-12 (Biochrom), 180 IU/ml Penicillin/Streptomycin and 0.375 μg/ml Amphotericin to maintain tissue viability. Utilizing 4 mm biopsy punches (Kai Medical GmbH), adipose tissue discs with a diameter of 4 mm and a thickness of approximately 1 mm were created, and randomly distributed to different culture conditions (MN = normal medium, MO = osteogenic medium) in Nunclon 24-well plates (Thermo Fisher Scientific).

### Gene transduction with adenovirus particles

Adenovirus encoding green fluorescent protein (Ad.GFP) or Adenovirus encoding h*BMP-2* (Ad.hBMP-2) (Sirion Biotech) with 1 × 10^8^ infectious units (IU) in 10 μl vector solution per adipose tissue disc was designed to be used for transduction. Hereby Ad.GFP was used to analyze the efficiency of transduction, in which the green fluorescent signals of transduced discs could be analyzed under a fluorescent microscope. After diluting the adenoviral particles in basic DMEM/Ham’s F-12, a volume of 10 µl vector solution was then pipetted directly on the surface of each punched adipose tissue disc. The transduced discs were then incubated in a Binder FED 115 incubator (BINDER) (set at 37 °C/5% CO_2_) for 1 hour. Non-transduced tissue fragments were treated in a similar way: 10 µl of DMEM/Ham’s F-12 were pipetted on the surface followed by incubation for 1 hour. The tissue pieces with vector particles were then transferred to 24-well plates, 4 tissue pieces per well, with normal growth medium and cultured for one day to permit for tissue acclimatization to culture conditions. Afterwards tissue discs were washed with 1x PBS to remove excess vector particles and then cultured under preset experimental conditions. Non-transduced and transduced tissue pieces were cultured in 24-well plates with 4 tissue pieces per well.

### Adenoviral transduction efficiency assay and quantitative analysis

In order to determine the Ad.hBMP-2 transduction efficiency within the adipose tissue, an Ad.GFP transduction experiment was performed. Briefly, 24 hours after transduction with Ad.GFP, transduced adipose tissue specimens were removed from the culture medium and washed with 1x PBS and then placed on Superfrost glass slides (Menzel). Non-transduced tissue discs were used as negative control. Adipose tissue specimens were then analyzed using LSM 880 laser-scanning confocal microscope (Zeiss) with a 5x air objective (Zeiss, 0.25 NA, WD = 12.5 mm), and with scanning depth set to 3–5 μm.

Quantitative analysis of transduction efficiency was performed utilizing the Image J software v.1.6 (NIH). The total area of the tissue specimens and the area occupied by GFP-positive cells within the tissue were calculated respectively. Nine samples per group were used for the analysis, and values were expressed as a mean percentage of GFP-positive cells occupied area in the total area of transduced tissue discs.

### Tissue culture

Adipose tissue samples were cultured with normal medium (MN) containing DMEM/Ham’s F-12, 10% FCS (Sigma–Aldrich), 60 IU/ml penicillin/streptomycin or osteogenic medium (MO) (DMEM/Ham’s F-12 + 10% FCS + 50 μM L-ascorbic acid 2-phosphate, 10 mM β-glycerophosphate, 10 nM dexamethasone). The media were changed every 3 days. The replaced media were collected and stored at −80 °C for protein analysis utilizing an ELISA assay. Adipose tissue specimens were cultured *in vitro* for 1, 2 and 4 weeks before being harvested for histological, qRT-PCR and immunochemical analysis.

### ELISA to detect hBMP-2 expression

In order to determine whether hBMP-2 was being expressed and to track the relative quantity of the protein over the designated culture period, an ELISA was performed. Briefly, by using a Quantikine ELISA Kit (R&D Systems), media were added into ELISA plates and hBMP-2 was then bound by pre-coated specific antibody. Afterwards an enzyme-linked monoclonal antibody specific for hBMP-2, followed by a substrate solution, were added to form an antibody-enzyme-substrate polymer. The color intensity of each well was measured using a Synergy HT microplate reader (BioTek). Media collected from the non-transduced adipose tissue discs cultured in normal medium and osteogenic medium served as controls. Nine samples per group were used for assay and measurements were performed in triplicate. The amount of BMP-2 produced by 4 adipose tissue specimen per well was presented.

### Histology

After 1, 2 and 4 weeks of culturing, the adipose tissue specimens were taken and fixed in 4% formalin for 24 hours and then processed into paraffin using a tissue processor STP-120 (Thomas-medical). Utilizing a Leica RM2255 microtome (Leica), sections were cut at the thickness of 10 µm from each adipose tissue specimen, and then stained using alizarin red S staining to identify calcium deposition in the tissue specimens. Hematoxylin and Eosin staining was also applied to detect the structure and components of adipose tissue.

### Histomorphometry

Images of the stained histological sections were captured using a M8 Microscope & Scanner (PreciPoint), with the ViewPoint Scanner software (PreciPoint). Histomorphometric analysis was then performed using the Image J software v.1.6 (NIH). The total area of the adipose tissue disc and the positive stained area generated within the tissue disc were established respectively. One representative section per tissue sample was analyzed. Nine tissue samples per group/condition were used for histomorphometry. Values were expressed as a mean percentage of positive stained area within the tissue discs.

### Quantitative reverse transcriptase polymerase chain reaction (qRT-PCR)

Total RNA extraction was performed on adipose tissue harvested for qRT-PCR analysis using a modified Trizol extraction method^51^. Four tissue discs cultured in each well were harvested as one sample for RNA isolation, and 9 samples per group were used for RNA isolation and qRT-PCR. qRT-PCR measurements were performed in triplicate. The concentration and purity of RNA were measured with a NanoDropTM Lite spectrophotometer (Thermo Scientific). Reverse transcription was subsequently performed using the QuantiTect Reverse Transcription-Kit (Qiagen).

Gene expression patterns of *Alkaline phosphatase* (*ALP*), *Osteocalcin* (*OCN*), *Bone sialoprotein* (*BSP)*, *Osteopontin* (*OPN)*, *Runt-related transcription factor 2* (*RUNX-2*), known markers that indicate bone formation, together with human *Bone morphogenetic protein 2* (h*BMP-2*) were analyzed utilizing qRT-PCR. Pre-designed primers for each gene were bought from Qiagen, on which all necessary pre-optimizations had been performed in advance. *Glyceraldehyde-3-phosphate dehydrogenase* (*GAPDH*) was chosen as the reference gene, with data from the various treatment tissue groups being normalized to the expression patterns of the endogenous untreated fresh adipose tissue control.

qRT-PCR was performed in triplicate using FastStart Essential DNA Green Master (Roche Diagnostics) with a Light Cycler 96 thermocycler (Roche Diagnostics). The reaction mixture contained 25 ng cDNA, 10 µM of each primer, 2x FastStart Essential DNA Green Master mix (Roche Diagnostics) in a final volume of 10 µl. Thermocycling parameters included a denaturation step of 95 °C for 10 min; 45 cycles of 95 °C for 10 s, 60 °C for 15 s and 72 °C for 15 s, with a final extension step at 37 °C for 5 min. Relative gene expressions were calculated using the 2^−ΔΔCt^ method and data represented as the normalized relative quantity (NRQ).

### Immunohistochemistry

Adipose tissue disc sections were immunohistochemically analyzed for OCN, OPN and Sclerostin (Scl) using the SuperVision 2 HRP single species kit (DCS Innovative). Briefly, tissue sections were deparaffinized and rehydrated through reducing gradients of ethanol into deionized water. Antigen was retrieved with EDTA buffer, pH 8.0 (DCS Innovative) in a 2100 Antigen Retriever (Aptum). After a 10 min incubation and 2 h cooling step, sections were washed with 1x PBS with 0.1% Brij (Sigma–Aldrich), followed by endogenous peroxidase activity blocking with 3% hydrogen peroxide (H_2_O_2_) (Merck) in 1xPBS. After washing, rabbit anti -rat primary antibodies for OCN, OPN and Scl (Biorbyt) were applied to the sections at a dilution of 1:100 and left to incubate for 1 h at RT. For negative controls, the primary antibody was omitted. Following primary antibody incubation, sections were incubated at RT with enhancer and polymer HRT, 20 min for each step with washing in between, after which 3.3′Diaminobenzidine (DAB) (DCS Innovative) was applied to the sections to visualize the proteins and counterstained with Mayer’s hematoxylin. Immunohistochemically stained sections were then analyzed under a M8 Microscope & Scanner (PreciPoint), and images were taken with ViewPoint Scanner software (PreciPoint).

### Immunofluorescent staining

Prior to immunofluorescent staining, the sections were firstly fixed with acetone (Applichem) for 15 minutes. Then 5% bovine serum albumin (BSA) (Sigma–Aldrich) was applied to block non-specific binding sites. Thereafter, primary antibody of hBMP-2 (rabbit anti-human) (Abcam) was added and incubated overnight at 4 °C. For negative control, the primary antibody was omitted. After intensive washing, sections were treated with 0.1% Triton X-100 solution (Sigma–Aldrich). The secondary antibody (goat anti-rabbit) (Jackson ImmunoResearch) was added and incubated for 30 minutes at RT. Thereafter, the nuclei were stained with Hoechst 33342 (Life Technologies). The slides were mounted with Fluoromount W (SERVA Electrophoresis), and allowed to dry in darkness at 4 °C. Microscopy was performed with the Zeiss Axioskop 40 equipped with AxioCam MRc 5 (Zeiss) and FluoArc (Zeiss). Images were obtained using Axio Vision, Rel. 4.9 software (Zeiss). Constant exposure time was assured for all the samples so that fluorescence intensity could be compared.

### Statistical analysis

Statistical analysis was performed using Prism 5.02 (GraphPad Software). Values were presented as mean ± standard error. Nine samples per group were used for all assays. Measurements were performed in triplicate. Statistical inferences were based on a paired t-test for two-group comparisons. The Mann-Whitney U-test was applied when the assumptions of a t-test were not met. Significances were set at p < 0.05, where *p < 0.05, **p < 0.01 and ***p < 0.001, indicating increases in significances.
